# Goats excel at learning and remembering a highly novel cognitive task

**DOI:** 10.1186/1742-9994-11-20

**Published:** 2014-03-26

**Authors:** Elodie F Briefer, Samaah Haque, Luigi Baciadonna, Alan G McElligott

**Affiliations:** 1 Biological and Experimental Psychology, School of Biological and Chemical Sciences, Queen Mary University of London, Mile End Road, London E1 4NS, UK; 2 Present Address: Institute of Agricultural Sciences, ETH Zürich, Universitätstrasse 2, 8092 Zürich, Switzerland

**Keywords:** Domestication, Long-term memory, Physical cognition, Social learning, Ungulates

## Abstract

**Introduction:**

The computational demands of sociality (maintaining group cohesion, reducing conflict) and ecological problems (extractive foraging, memorizing resource locations) are the main drivers proposed to explain the evolution cognition. Different predictions follow, about whether animals would preferentially learn new tasks socially or not, but the prevalent view today is that intelligent species should excel at social learning. However, the predictions were originally used to explain primate cognition, and studies of species with relatively smaller brains are rare. By contrast, domestication has often led to a decrease in brain size, which could affect cognition. In domestic animals, the relaxed selection pressures compared to a wild environment could have led to reduced social and physical cognition. Goats possess several features commonly associated with advanced cognition, such as successful colonization of new environments and complex fission-fusion societies. Here, we assessed goat social and physical cognition as well as long-term memory of a complex two-step foraging task (food box cognitive challenge), in order to investigate some of the main selection pressures thought to affect the evolution of ungulate cognition.

**Results:**

The majority of trained goats (9/12) successfully learned the task quickly; on average, within 12 trials. After intervals of up to 10 months, they solved the task within two minutes, indicating excellent long-term memory. The goats did not learn the task faster after observing a demonstrator than if they did not have that opportunity. This indicates that they learned through individual rather than social learning.

**Conclusions:**

The individual learning abilities and long-term memory of goats highlighted in our study suggest that domestication has not affected goat physical cognition. However, these cognitive abilities contrast with the apparent lack of social learning, suggesting that relatively intelligent species do not always preferentially learn socially. We propose that goat cognition, and maybe more generally ungulate cognition, is mainly driven by the need to forage efficiently in harsh environments and feed on plants that are difficult to access and to process, more than by the computational demands of sociality. Our results could also explain why goats are so successful at colonizing new environments.

## Introduction

Several hypotheses have been proposed to explain the evolution of cognition; the two main ones being the “Machiavellian intelligence” [1] or “social brain or intelligence hypothesis” [2,3] and the “ecological competence” or “behavioral flexibility hypothesis” [4,5]. These hypotheses both assume that large and costly brains have evolved in order to confer higher cognitive capacities, but they differ in the main selection pressures that could have driven the evolution of cognition. The social intelligence hypothesis suggests that sociality, which arises from the need to minimize predation risk, involves strong computational demands (e.g. maintaining group cohesion, coordination and reducing conflict). According to this hypothesis, the demands of group living are the main initial driver responsible for the evolution of cognition. Correlational studies have indeed found that species that form social associations have larger brains than solitary ones, in various orders of mammals characterized by small and large brain sizes (e.g. primates, carnivores and ungulates [6]). One consequence of group living is that individuals have increased opportunities to learn from each other, for example how and where to forage [7]. The social intelligence hypothesis thus also assumes that ecological problems are mainly solved socially [8]. Social learning is a potentially less costly and faster way to acquire adaptive information than individual learning, implying that it should have been favored throughout the evolution of cognition [9,10].

Alternatively, the behavioral flexibility hypothesis proposes that it is the skills required to solve complex ecological problems (e.g. extractive foraging, memorizing resource locations) that lead to the evolution of cognition. Accordingly, innovation and tool use (among other traits), are correlated with large brains in birds and primates [4,11]. Unlike the social intelligence hypothesis, the behavioral flexibility hypothesis proposes that ecological problems are mainly solved individually, and that it is the need for individual problem solving abilities that lead to the evolution of cognition [4,8]. Alternatively, social and asocial learning abilities could also overlap and be positively correlated [11], if general cognitive skills are initially acquired under the influence of social role models [12], or if they are based on the same mechanisms [13]. The prevalent view today of the evolution of cognition, is that social species that are intelligent should have good social learning abilities. Yet, is this the case for all species, even non-primates and smaller-brained species? Studies investigating the evolution of cognition tend to focus on species characterised by relatively large brains and complex cognition (e.g. primates and birds; [14,15]), although this bias has decreased in recent years [16]. The cognitive abilities and factors affecting cognition in species that are characterised by smaller relative brain sizes (e.g. ungulates), have received far less attention.

Domestication is known to strongly affect brain size. Consistent reductions in brain size relative to body size, as well as in brain size parts, have occurred in many domestic species [17], even when comparing wild and domesticated animals of comparable body size and age (e.g. wild cavies and guinea pigs, *Cavia aperea*[18], and wild and farmed American mink, *Mustela vison*[19]). The largest reductions have occurred in the limbic system, which controls emotionally guided behaviors and memory [20]. These changes presumably result from intense selection during the domestication process for reduced wariness and low reactivity to external stimuli [21]. The assumption that larger brains confer better cognitive abilities has not yet been clearly empirically demonstrated [22]. Furthermore, the reduction in relative brain to body size with domestication could be largely due (at least in some species), to selection for larger body size, rather than to reduction in brain size per se [23]. However, if reduction in brain size impacts on cognition, we might expect domestic species to show reduced cognitive abilities compared to their wild relatives [20]. Related to this is the common public perception that domestic ungulates, such as sheep (*Ovis aries*) and goats (*Capra hircus*) are not intelligent.

Domestication is a process of increased dependency between humans and domestic animals. This process leads to an adaptation of the animals to a captive environment through both natural and artificial selection inducing genetic changes occurring over generations [21]. It is accompanied by modifications in behavior, physiology and morphology, which can be actively selected by humans, or simply be by-products of this selection or consequences of captivity (e.g. stable and predictable environment, low risk of predation, easily accessible food resources, frequent interactions with humans; [24,25]). The effects of domestication on cognition have mostly been studied by comparing dogs (*Canis familiaris*) and wolves (*Canis lupus*). These comparisons have revealed that, despite some controversy [26,27], dogs could have reduced physical cognition abilities compared to wolves [28]. They could also have enhanced abilities to learn from humans (i.e. heterospecific social learning; “domestication hypothesis”) [29,30], but reduced abilities to learn from conspecific as a result of relaxed dependency on conspecifics during domestication [31].

Dogs have mainly been domesticated as hunting companions and pets [32], implying good socio-cognitive abilities for dog-human communication [30], through direct selection on these abilities or by-product of selection on other traits of interest [33]. By contrast, most ungulates have been domesticated for milk, meat and hair production [21], which do not imply any active selection on cognitive traits. Surprisingly, with the exception of studies on pigs and wild boars (*Sus scrofa*) [34], the effect of domestication on ungulate cognition has been very poorly studied. Although ideally, the responses of wild and domestic animals to similar tests would have to be compared, using wild animals is often impossible because they are not habituated to humans and therefore their high stress responses lead to decreased performance compared with their domestic counterparts [20]. Therefore, it is crucial to first acquire detailed knowledge on the cognitive performance of domestic animals, in order to find what their cognitive limitations are, and what would be interesting to test in their wild counterparts for comparisons.

In this study, we tested social and physical cognition as well as long-term memory of a domestic ungulate, the goat (*Capra hircus*), to investigate the selection pressures affecting the evolution of cognition in this species. More specifically, we tested 1) if, despite domestication, goats can learn a complex foraging task (two-step food box) and if they have long-term memory of this task and 2) if the cognitive abilities shown in (1) are necessarily accompanied by social learning skills (i.e. with the ability to learn the foraging task socially). Domestic and feral goats possess several features commonly associated with advanced cognition [15] (for example, successful at colonizing new environments, varied diet, long life, good visual acuity and complex fission-fusion societies [35-38]). Goats have a movable, split upper lip enabling them to manipulate objects. They are highly selective browsers and grazers, which thrive in many different, harsh environments [39,40]. They are often faced with situations in which food is difficult to access and to process (e.g. extract leaves from among hard thorns on woody legumes), particularly when feeding in mountainous or arid areas [35,41]. Furthermore, they live in large, complex social groups (fission–fusion societies [42]). All these ecological factors suggest that they could benefit from good social and physical cognitive abilities during foraging. However, probably as a result of domestication, domestic goats have smaller brain mass (0.13 kg) than wild goats (*Capra aegagrus*; 0.18 kg), despite having larger body mass (body mass: domestic goat, 80.0 kg; wild goats, 45 kg; [43]).

According to the domestication hypothesis, because domestic animals depend on humans to obtain food, it is possible that their abilities to forage and extract food, as well as to remember food location and extraction techniques are reduced [44,29]. In this case, we would expect domestic goats not to be able to manipulate and learn our foraging task and/or not to be able to remember it after long time intervals. The same predictions would apply if the decrease in brain size linked to domestication impacts on cognitive abilities. According to the social intelligence hypothesis, goats should preferentially learn the task using social learning. In this case, we would expect the goats that learn the task after observing a conspecific demonstrator to learn faster than if they do not have that opportunity [45]. However, domestic animals could also rely on conspecifics less than their wild counterparts and show reduced social learning abilities [31]. This type of experimental study can provide a much greater understanding of the effect of domestication on cognition, and in particular on social intelligence in domestic species that have been actively selected for physical (e.g. meat or milk production) and not cognitive traits (e.g. good communication skills).

## Results and discussion

### Social and physical cognition

**Figure 1:**
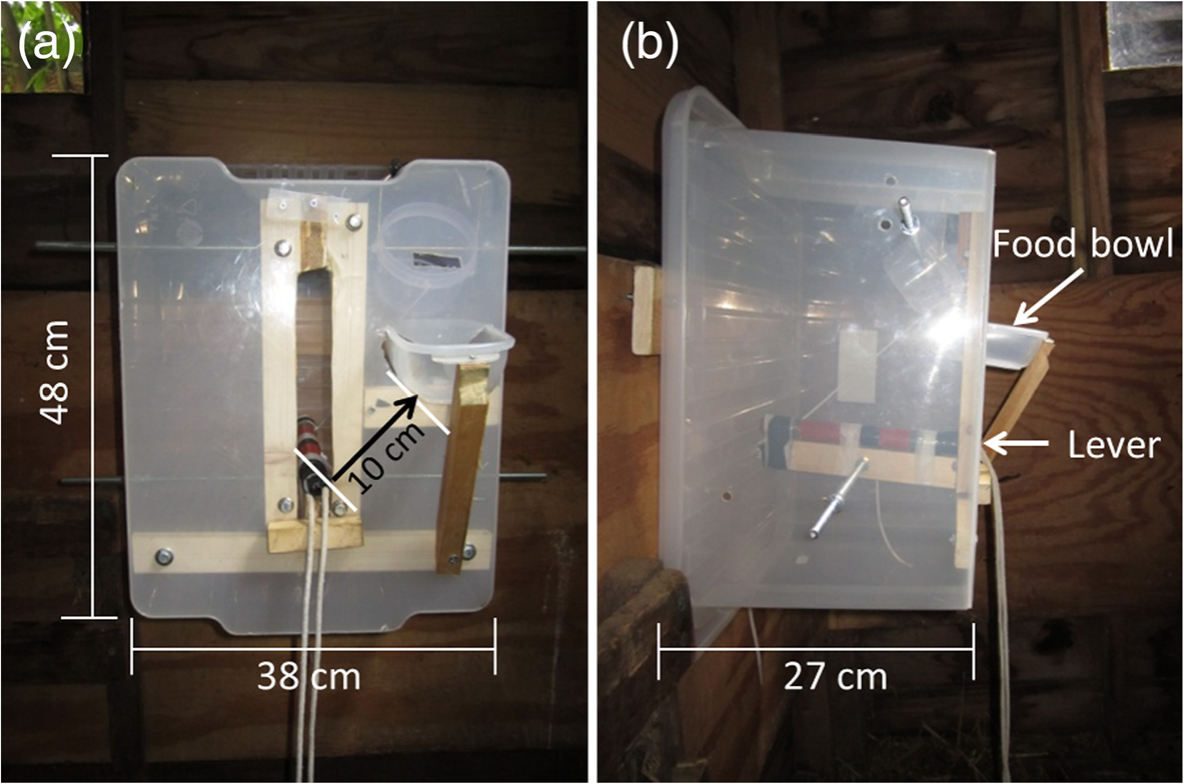
**Two-step food box used in the experiments. (a)** Frontal view and **(b)** lateral view of the box before the trial, showing the dimensions and the bowl where the food was falling after actionning the lever.

**Figure 2:**
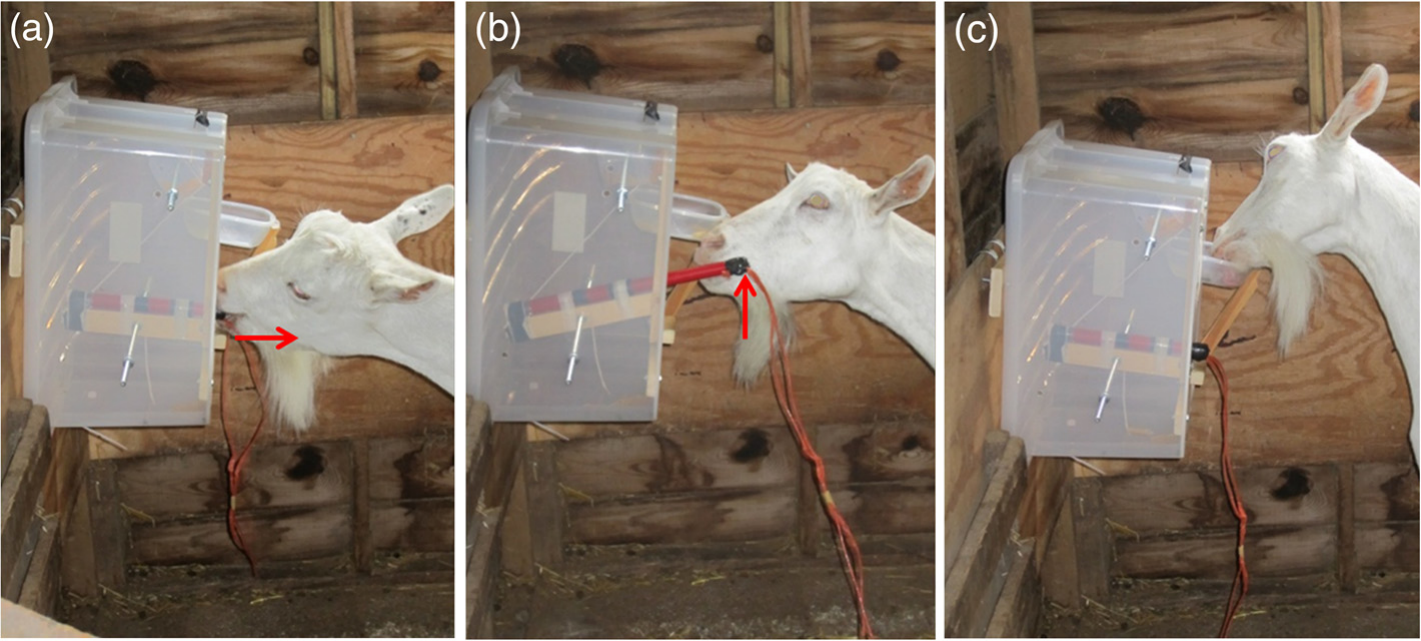
**Two-step task.** One goat demonstrating **(a)** the first step (“pull lever”), **(b)** the second step (“lift lever”), and **(c)** eating the reward. Red arrows indicate the direction required to complete the action (see also Additional file 1).

To assess whether goats can learn a complex task, and if they use social learning to this aim, we used an adaptation of the “artificial fruit challenge” developed for nonhuman primate research [46,47] (Figures 1 and 2). It allowed us to test for the potential existence of both simple (e.g. stimulus or local enhancement) and complex forms of social learning (e.g. emulation or production imitation), which have not been reported in ungulates [45].The artificial fruit is a box that contains food, which can be accessed through a certain procedure. Social learning is evident when animals learn faster with than without a demonstrator [48], or if they use the same solution as the demonstrator more often than alternatives, in case the food can be accessed through more than one solution [46]. Similar food boxes have also been used to test for innovation and problem solving abilities in birds [49], primates [50] and carnivores [51,52] (“food extraction tasks” or “puzzle boxes’’). Similarly, as in van de Waal and Bshary [47] and Caldwell and Whiten [48], our box contained food that could be accessed through one solution, but which consisted of a sequence of actions (two-steps). Goats had to pull out a lever with their lips or teeth using a rope (step 1; “pull lever step”). They then had to lift the lever up using the mouth or muzzle (step 2; “lift lever step”), which made a food reward drop from a dispenser into a feeding bowl (Figure 2; Additional file 1). Our study animals were trained to use the device with (“observers”; *N* = 4) or without (“controls”; *N* = 3) a demonstrator goat (*N* = 5; *N* = 12 goats originally trained in total; Table 1). We predicted that if goats have good physical cognition abilities, they would be able to manipulate the box and learn this task. We also predicted that if they use any form of social learning, they would be faster at learning after observing a demonstrator, than if they did not have that opportunity.

**Table 1:** Characteristics of the goats and number of trials to success

**Goat**	**Breed**	**Horn**	**Sex**	**Age**	**Category**	**Number of trials**
1	British Toggenburg	No	Male	7	Demonstrator	11
2	British Saanen	No	Male	15	Demonstrator	13
3	British Saanen	No	Female	7	Demonstrator	22
4	Pygmy	Yes	Male	8	Demonstrator	*Removed trial 7*
5	British Alpine	Yes	Female	5	Demonstrator	12
6	Golden Guernsey	No	Male	10	Observer	10
7	British Toggenburg	Yes	Female	7	Observer	13
8	British Toggenburg	Yes	Male	7	Observer	*Removed trial 12*
9	British Alpine	Yes	Female	7	Observer	*Lift-lever up step:* 10
10	British Toggenburg	No	Male	9	Control	10
11	Golden Guernsey	No	Male	6	Control	9
12	Anglo Nubian	No	Female	11	Control	8

Characteristics of the goats used in the experiment as demonstrators, observers or controls (additional goats tested that were not demonstrators or observers), along with breed, presence of horns, sex and age. The number of trials required to successfully learn the two-step task is indicated. Two goats (4 and 8) were removed from the test because they used an alternative method (horns) to try to obtain the reward and did not learn the lift-lever step. One goat (9) learned the lift-lever step within 10 trials, but did not learn the whole task (two-steps) within 22 trials.

All goats (demonstrators, observers and controls) were trained to perform the two-step task in the same way, using a shaping procedure, starting with the lift-lever step (i.e. the lever was already pulled out at the beginning of each trial and the goats had to lift it up to obtain the reward) and then the two steps in a row (i.e. the lever was inside and the goats had to perform the two steps; Additional file 2). Observers were exposed to a clearly visible demonstrator goat in an adjacent stable (“model–observer dyad” [48]), performing the task three times, before every learning trial (from trial 1 to success).

In total, 9/12 trained goats successfully learned the two-step task. This was done within 12.0 ± 1.4 trials (range = 8–22 trials; corresponding to 4.33 ± 0.54 days of training for the entire learning phase, range = 3–6 days; Table 1). Among these successful goats, the number of trials required to learn the task did not differ between goats that had a demonstrator (two observers: 11.5 ± 1.5 trials) and those that did not (four demonstrators and three controls: 12.1 ± 1.8 trials; linear model (LM): *F*_1,1_ = 0.96; *P* = 0.51), suggesting that goats did not learn the task socially. Because of our small sample size, we carried out a power analysis to calculate the significance level to use in order to obtain a power of 0.8 with our sample size. This analysis revealed that the significance level would have to be raised to α = 0.32 (instead of 0.05), which is still lower than the p-value we found for the effect of the demonstrator presence (*P* = 0.51).

Ten of the 12 tested goats successfully learned the lift-lever step within 8.0 ± 0.6 trials (range = 5–10 trials). The number of trials taken by observers to learn this step (9.0 ± 1.0 trials; *N* = 3 goats) did not differ from the number of trials taken by goats that did not have a demonstrator (7.6 ± 0.6 trials; *N* = 7 goats; LM: *F*_1,2_ = 0.07; *P* = 0.82). A power analysis revealed that the significance level would have to be raised to α = 0.32 to obtain a power of 0.8, which is still lower than the p-value we found for the effect of the demonstrator presence (*P* = 0.82). While learning the lift-lever step, goats lifted the lever for the first time after 4.4 ± 0.3 trials (range = 3–7).

Nine goats successfully learned to perform the two steps consecutively after 4.2 ± 1.2 additional trials (range = 3 trials for all goats except one with 14 trials). There was no effect of the presence of a demonstrator (3.0 ± 0.0 trials; *N* = 2 goats) or not (4.6 ± 1.6 trials; *N* = 7 goats; LM: *F*_1,1_ = 0.19; *P* = 0.74) on this number of trials. A power analysis revealed that the significance level would have to be raised to α = 0.40 to obtain a power of 0.8, which is still lower than the p-value we found for the effect of the demonstrator presence (*P* = 0.74). While learning the whole sequence of steps, goats performed the two steps for the first time after 1.7 ± 0.6 trials. There was no significant effect of age (LM: *P* ≥ 0.26 in all cases), sex (*P* ≥ 0.26 in all cases) or breed (LM: *P* ≥ 0.18 in all cases) on any of the parameters (number of trials to success, to learn to perform the lift-lever step and to learn to perform the two steps consecutively).

One of the 12 originally tested goats (goat 9 in Table 1), who was an observer, succeeded in the lift-lever step in 10 trials, but was still unable to complete the two-step sequence by trial 22 and showed no signs of improvement. She was not used in any further memory tests. Two other goats (one observer and one demonstrator; goats 4 and 8 in Table 1), were removed from the experiments after the first 7–12 trials, because they tried to use their horns instead of their muzzle to get the reward and therefore risked damaging the box.

Although learning was assisted, most of the goats (9/12) were able to learn the task, suggesting good physical cognition abilities that allowed them to manipulate objects. However, we did not find evidence for social learning. From a total of four goats originally tested with a demonstrator (i.e. observers; goats 6-9 in Table 1), one of them used an alternative method (horns) to try to obtain the reward, and had to be removed from the experiment (goat 8 in Table 1). Another one learned the lift-lever step, but never learned the other step (goat 9 in Table 1). The remaining two goats did not learn faster than goats trained without a demonstrator. This absence of evidence for social learning could be due to the technical difficulty of our task. In some primates, social learning of step sequences revealed few (2/14 [53]; 2/12 [47]) or even no success [48] in opening a food box, suggesting that this is a cognitively demanding form of social learning that might not occur in goats. However, we could have found evidence for simpler social learning forms, such as local enhancement (i.e. observers would be attracted to the location of the box, immediately after the demonstrator interacted with it), or stimulus enhancement (i.e. observers would be attracted to the box itself as well as to similar objects, even when the demonstrator is not present [45]). Our results therefore confirm previous findings suggesting a lack of social learning by observation in goats [54].

Goats are highly social animals that live in fission-fusion societies, with groups varying in size throughout the day and aggregating in permanent night camps in the evening [42,55]. They have been shown to use some form of social information; they follow conspecific gaze direction and human pointing, but not human gaze [56]. There is also evidence for perspective taking, as goats that receive aggression from dominant individuals prefer to eat food that dominants cannot see [57]. These show that they understand dominance relationships [37], and that they can use some basic forms of social learning. However, it is possible that the delay between the removal of the demonstrator and the training of the observer in our experiment was too long. Goats might be capable of local enhancement (i.e. they would be attracted to a demonstrator’s location while it is still present or immediately after it had been removed from that location), but not of “delayed local enhancement”. This social learning mechanism is a form of “stimulus enhancement” that would lead an individual to be attracted to the location of a demonstrator, for more than a short period after it has been removed [45]. The absence of delayed local enhancement in goats is consistent with results of our previous research, showing that goats do not copy the side to which the demonstrator goes to feed after it has been removed from the experimental apparatus [54]. Similar lack of social learning have been found with horses (*Equus caballus*) in studies in which observers were tested much later after demonstrators had been removed from the experimental arena (on different days), [58,59]. However, in tests when observer horses were tested immediately after the demonstration, social learning did occur [60,61]. In Krueger and Flauger [62], the side at which a demonstrator was eating influenced the behaviour of observers if the demonstrator was present (i.e. observers avoided the side of the demonstrator), but not if the demonstrator was out of sight. We suggest that wild and domestic goats, and possibly other ungulates, lack sophisticated forms of social learning, because individual and simple social learning mechanisms are probably more efficient for improving their survival or reproductive success [45]. Alternatively, this lack of social learning could be linked to domestication, following the relaxed dependency on conspecifics, as has been suggested for dogs [31].

### Long-term memory

To assess environmental intelligence, we measured long-term memory of the food box task. Memorizing information for extended periods of time (e.g. weeks or years) is limited by the size of brain parts, such as the hippocampus, suggesting that this process is costly in terms of brain tissue [63]. We tested if all the goats successfully trained on the two-step task (with or without a demonstrator; *N* = 9 goats), remembered it after retention intervals ranging from 1 month to 9–10 months. We predicted that if goats had long-term memory of the task, their latency to solve it after extended time intervals should be much shorter than the duration of the initial training, and should not increase with time. To our knowledge, long-term memory for obtaining a food reward from “artificial fruit” has not been reported for any species.

We tested the memory of the goats with a first test 26–33 days after the end of the learning phase (0.9-1.1 months; *N* = 9 goats), and a second test 281–311 days after the first memory test (9.2-10.2 months; *N* = 8 goats). One goat was tested at an intermediate stage of 168 days, followed by another test 139 days after her first test. All the goats solved the task within one trial. During the first memory test, all 9 goats solved the two-step task within 35.78 ± 7.95 s (range = 12–91 s; after 0.9-1.1 months of retention). During the second test, it took them 38.75 ± 9.38 s to solve the task (range = 6–72 s; after 9.2-10.2 months of retention; *N* = 8 goats). The goat tested during the intermediate memory tests took 4 s (after 168 days of retention) and 6 s (after 139 days of retention), respectively, to solve the task.

**Figure 3:**
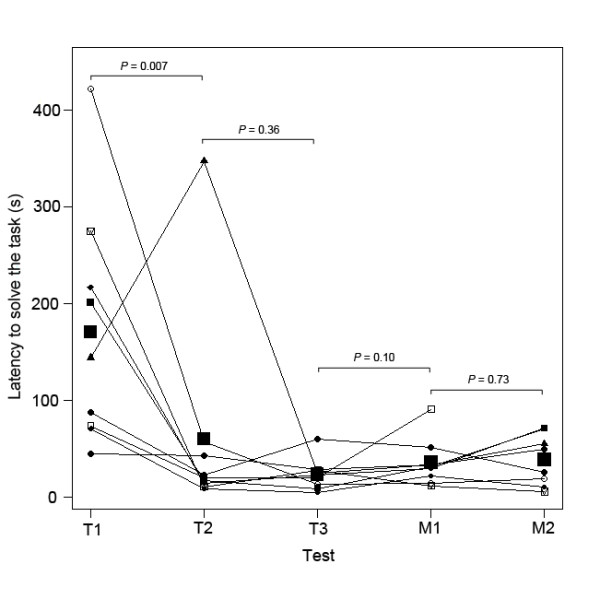
**Latency to solve the two-step task during the training phase and memory tests.** Dots represent the latency for each individual to perform the two-steps during the three last trials of the learning phase (T1 = third-to-last trial (first successful trial); T2 = second-to-last trial (second successful trial); T3 = last trial (third successful trial)) and the two memory tests (M1 = first memory test after 26–33 days of retention; M2 = second memory test after 281–311 days of retention). Lines show repeated measures of the same individual across tests. Large black squares indicate the mean latency for each test. Latencies differed between T1 and T2, but not between T2 and T3, T3 and M1 or M1 and M2 (Linear mixed effects models).

**Figure 4:**
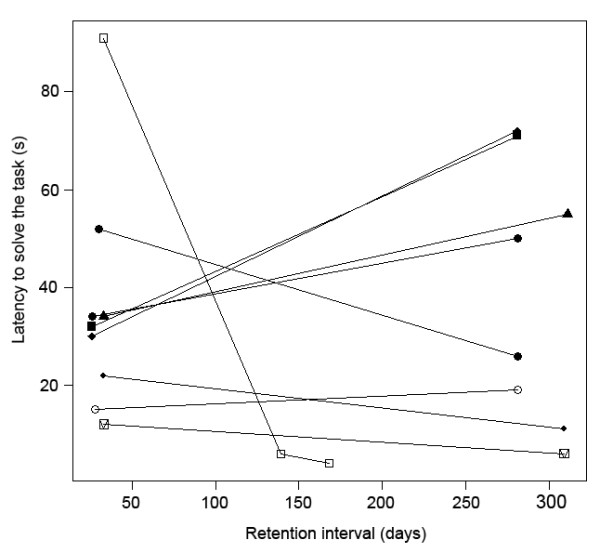
**Latency to solve the two-step task as a function of the retention interval.** Individuals are indicated by different shapes. Lines show repeated measures of the same individual across test. There was no effect of the time elapsed since the last test on how quickly goats solved the task. For each memory test, all the goats that had successfully learned the task solved it within one trial and less than 2 minutes.

Additionally, we compared the latency to solve the task during the three last trials of the learning phase (successful trials required to reach the learning criterion; third-to-last learning trial, “T1”; second-to-last learning trial,“T2”; last learning trial, “T3”) with the latency to remember the task during the two memory tests (first memory test, “M1”; second memory test, “M2”; Figure 3). These analyses showed that the latency to perform the two-step task differed according to the type of test (T1-M2; linear mixed effect model: *F*_1,31_ = 8.20, *P* = 0.0001). It decreased between T1 and T2 and then stayed constant between T2 and T3, T3 and M1, and M1 and M2 (Figure 3). This pattern is consistent with an increase in performance over the three last trials of the learning phase, followed by long-term memory of the task between T3, M1 and M2. Furthermore, there was no effect of the retention interval (range 26–311 days) on the latency to solve the task (linear mixed-effect model: *F*_1,9_ = 0.03; *P* = 0.87; Figure 4). A power analysis revealed that the significance level would have to be raised to α = 0.76 to obtain a power of 0.8, which is still lower than the p-value we found for the effect of the retention interval (*P* = 0.87). This absence of increase in the time needed to solve the task with increasing retention intervals indicates good long-term memory. There was no significant effect of age (LM: *P* ≥ 0.18 in all cases), sex (*P* ≥ 0.40 in all cases) or breed (LM: *P* ≥ 0.22 in all cases) on any of the parameters (time to solve the task during the last trials of the learning phase or during the memory tests).

Although during the learning phase, goats lifted the lever up for the first time after an average of 4.4 trials, and performed the two steps for the first time after 1.7 additional trial, their performances (1 trial of 4–91 s to solve the task) over extended intervals (1–10 months) indicates that they very quickly remembered how to obtain food from the box. There was no increase in the time needed to solve the task with increasing retention intervals, suggesting that the goats retained precise information. Previous studies have shown that horses have exceptional long-term memory of 10 years for size concepts [64]. Similar findings have been reported in California sea lions (*Zalophus californianus*) that remember identity concepts [65], and in elephants (*Loxodonta africana*) that can remember family member vocalisations for at least 12 years [66]. However, those memories could be considered to be for more naturally occurring information in the environment. Our results present the first evidence for long-term memory of a highly novel and artificial food box. In a recent study, we showed that mother goats have long-term memory for the calls of their own kids (at least 13 months after separation [67]). Goats can also remember visual shapes for 42 days [68]. Here, we show that their remarkable long-term memory can be generalised across novel cognitive challenges. Finding food sources that are ephemeral and widely distributed in clumps requires more cognitive abilities to obtain than foods that are available all year and at the same locations [69]. Feral goats have colonised a large variety of novel, harsh environments worldwide, often becoming pests as a result [35]. The cognitive skills revealed in our study help explain why goats are so adaptable.

The mechanisms behind the long-term memory of the food box shown in our study are unclear. The most probable explanation would be that the process involved is recognition of the box (i.e. association of an object with one previously encountered) followed by procedural memory of the task (i.e. unconscious memory of skills), as opposed to recall, which would involve remembering how to solve the task in the absence of the box [70]. Further tests could be designed to investigate the exact mechanisms involved. In addition, comparisons between our results and cognitive skills found in wild goats would provide useful knowledge about the impact of domestication on ungulate cognition. Wild goats might be better problem solver and/or social learner than domestic goats, if the reduced natural selection pressures linked to domestication (e.g. easily available food, low or non-existent predator risk, relaxed dependency on conspecifics), compared to a wild environment, have resulted in a lower need for these cognitive abilities [21]. Alternatively, domestic goats could be better problem solver and/or social learner than wild goats if domestication results in a reduction in neophobia and thus in an increase in exploratory behavior [51,61,71].

## Conclusions

We assessed the potential selection pressures behind the evolution of ungulate cognition, by testing social and physical cognition as well as long-term memory of a two-step task. Nine of twelve originally trained goats (75%) successfully learned the task within 8–22 trials. During the memory tests, all nine solved the task considerably faster than during the first exposures (< 2 min), indicating long-term memory. The presence of a demonstrator did not decrease the number of trials required to learn the task, suggesting that goats did not use social learning. Therefore, we propose that goat cognition could have been driven by the need to survive in harsh environments, more than by the computational demands of sociality. Our results could also explain why goats are so successful at colonizing new environments. Although comparisons with wild goats would be needed, our results indicate that goat domestication has not decreased their physical cognition abilities, but maybe their social learning skills. This challenges the common belief that domestic ungulates have low cognitive abilities and that clever animals necessarily have good social learning abilities. We propose that more experimental studies of species with relatively smaller brains should be carried out to gain a more thorough understanding of the evolution of cognition.

## Materials and methods

### Subjects and management conditions

The study was carried out at Buttercups Sanctuary for Goats, Kent, UK. We tested 12 adult goats (7 males and 5 females of various breeds; Table 1). The test animals were habituated to human presence and handling and had been at the sanctuary for at least one year. These goats were previously used for other experiments on emotions and temperament (e.g. [72]), but they had not been used for any other physical cognition tests and were therefore completely untrained at the beginning of the study.

Routine care of the study animals was provided by sanctuary employees and volunteers. During the day, all goats were released together into one of two large fields. At night, they were kept indoors in individual or shared pens (2 or 3 goats, average size = 3.5 m^2^) with straw bedding, within a larger stable complex. Goats had ad libitum access to hay, grass (during the day) and water and were also fed with a commercial concentrate in quantities according to their state and age. They also received fruits and vegetables on a daily basis. The research plan for our study was reviewed by the UK Government Home Office inspector for Queen Mary, University of London.

### Experimental design

We designed and built a transparent plastic food box (38 × 27 cm and 48 cm high; Figure 1), which could easily be manipulated by goats using their mouth or muzzle. The box contained a preferred food reward (a small handful of dry penne pasta and a few grass pellets), which could be accessed in two steps (pull-lever and lift-lever steps). These steps are described in Figure 2 (see also movie in Additional file 1). It was necessary for the goats to carry out the first step, in order for the lever to be released, so that they could then raise it, resulting in the food dropping into the bowl. A small slot was made near the food area to allow the goats to smell the food and to attract their attention to the box at the beginning of the learning phase. The box was fixed to a wall at 1 m above the ground (70 cm for the pygmy goat), within a pen (2 × 2.26 m). Goats were tested individually to avoid aggressive interactions at the box, which would potentially have prevented low-ranking subjects from participating [73]. All tests were video recorded using a Sony DCR-SX33E camcorder.

### General protocol

All goats received an initial training phase. The individuals that successfully learned the task during the training phase (9/12 goats) were then subjected to two memory tests after long retention intervals, during which they had not interacted with the food box at all (26 to 311 days). Goats were attributed to the “demonstrator” category (i.e. trained and subsequently used for demonstration to observers), “observer” category (i.e. trained in the same way as the demonstrator, but with a demonstration before every trial), or “control” category (i.e. trained in the same way as the demonstrator, without any demonstration). Initially, five demonstrator goats (goats 1–5 in Table 1) were trained in July 2011. Four of these five goats successfully learned the task (goats 1, 2, 3 and 5 in Table 1). Their first memory test occurred in August 2011. They were then used as demonstrators during the learning phase for four observer goats (goats 6–9 in Table 1) during August and September 2011. Three new control goats were also trained without a demonstrator at the same time, to test for the influence of social information (goats 10–12 in Table 1). The successful observer (2/4 goats) and control goats (3/3 goats) had their first memory test during October 2011. The second memory test occurred for all the goats during July 2012. The test for intermediate memory was carried out on one goat during February 2012.

### Learning phase

The learning phase was the same for all goats (demonstrators, observers and controls), with the only difference that, before every trial, observers watched a demonstrator performing the task (see details in below in *Social learning test*). During the first exposures to the box, none of the goats showed any particular motivation to interact with it, possibly because they had never previously been used in other physical cognition tests, and had thus not formed any associations between the box and a food reward [74,75]. Furthermore, they did not interact with the lever, which was situated 10 cm away from the feeding bowl (Figure 1). To manipulate the lever, goats had to inhibit their tendency to try to grab the food through the transparent sides of the box [52,76]. For these reasons, we decided to shape learning by dividing the learning phase into several stages. This allowed us to facilitate the learning of the two-step task by directing the goat’s attention towards relevant parts of the box (Additional file 2).

The first two stages were aimed at habituating the goats to the experimental setup and to show them where the reward was located. Because none of the goats interacted with the lever at the end of the second stage, we decided to split the learning phase into two further stages; one (third stage) in which the lever was already pulled out and the goats had to lift it up to obtain the reward (lift-lever step), followed by another stage (fourth stage) in which the lever was inside and the goats had to perform the two steps of the task (pull-lever and lift-lever steps) to obtain the reward. We assumed that goats had successfully learned the step (lift-lever steps) or task (two steps of the task) when they succeeded in obtaining the reward three times in a row, for three consecutive trials. Eight out of 10 goats (2 of the original 12 goats had to be removed from the experiment) completed the fourth stage within 14 trials or less. If the tested goat had not reached the learning criterion by trial 14 (*N* = 2 goats; goats 3 and 9 in Table 1), a piece of pasta was threaded onto the rope to encourage the goats to pull out the lever by chance after biting the rope (fifth stage). One of these two goats (goat 9 in Table 1) failed to learn the task after 22 trials and she was not included in further experiments. Goats received 1–7 trials per day (mean ± standard error (SE) = 2.69 ± 0.17) depending on the learning stage.

After the goats reached the learning criterion of three successful completion of the two-step task for three consecutive trials, they were not tested again until the memory tests, with the exception of the demonstrator goats. Once the first of the four trained demonstrators had successfully learned the task, they had one reminder trial, which consisted of letting them solve the task three times, once per day until all the other demonstrators had learned the task. One month later, these goats had their first memory test, before demonstrating the task to the observers.

### Social learning test

To test whether goats could use social learning to solve the two step task, four observers were trained as described above, in the same way as the demonstrators and controls (*Learning phase;* i.e. using a shaping procedure; Additional file 2), except that they were exposed to a demonstrator goat performing the task before every trial (“model–observer dyad” [77]).

Each observer (*N* = 4) was paired with a demonstrator goat (*N* = 4) that shared its pen at night (“stable mate”). We therefore considered observer-demonstrator pairs to be socially compatible and unlikely to show aggression [73]. The four demonstrators were used for the social learning test after they had been successfully trained with the task and after their memory had been tested one month later. They were placed in the pen with the food box, before every single trial of the observer (trial 1 to success), while being watched by the observer from the adjacent pen (2.58 × 2.26 m). Two experimenters monitored the behavior of the observer, ensuring that it viewed the demonstrator (defined as looking towards the demonstrator with ears pointing forward) performing the task three times in a row. Afterwards, the demonstrator was taken out of the pen and the observer’s trial started. The rest of the learning phase procedure was identical to the demonstrators (see *Learning phase*). The three control goats were also trained at the same time, following the learning procedure described above, but without a demonstrator.

### Memory tests

Testing consisted of a first memory test after 26–33 days (0.9-1.1 months) of retention (i.e. after the last contact with the box; *N* = 9 goats), and a second memory test after another 281–311 days (9.2-10.2 months) of retention (*N* = 8 goats). One of the goats was tested during an intermediate memory test after 168 days (5.5 months; goat 5 in Table 1) to verify that goats were still able to solve the task after such a long retention interval. This goat was tested again during the second memory test, after 139 days (4.6 months) without any contact with the box.

During each memory test, the tested goat was placed in the pen containing the box with a small amount of pasta and grass pellets in the bowl. The test ended when the goat solved the two-step task and obtained the food reward (maximum trial duration = 10 min). Using the videos of the three last trials of the learning phase (i.e. first to third successful trials) and the memory tests, we measured the latency to solve the two-step task. Latencies were defined as the time from when goats started to interact with the box by touching the rope or lever with their muzzle or pushing the box with their head, until they performed the two steps of the task and obtained the food. Occasionally, the goats pushed the bowl inside the box with their heads, in which case an experimenter would approach the box and readjust it into the correct position. The latency measure excluded the time when the experimenter was present. It also excluded the time spent eating from the bowl, defined as when the goat had its muzzle in the food bowl, and the time when the goat was looking away from the box (e.g. searching for food on the ground).

### Statistical analyses

Because of the limited sample size, demonstrators (*N* = 4) and successful controls (i.e. goats neither used as demonstrator, nor as observer; *N* = 3), which had been trained in exactly the same way, were grouped together (i.e. *N* = 7 goats trained without a demonstrator) and compared to observers, in order to test for social learning. We analyzed the data from the learning phase and memory tests using linear models and linear mixed-effects model (lm and lme functions in R [78]).

First, we investigated, using linear models (lm function in R), the effect of the presence of a demonstrator (fixed effect) and of the breed, age and sex of the goats (fixed control factors) on (dependent variable): model 1) the number of trials required to learn the lift-lever step (up to third stage of learning phase); model 2) the number of trials required to learn the whole task (two steps). Because we had one value per individual in each of these four models, we did not control for individual identity.

We then carried out linear mixed-effects models (LMM, lme functions in R) to compare the latency to solve the task during the three last trials of the learning phase (i.e. first to third successful trials) and during the memory tests. We also tested the effect of the retention interval on the latency to solve the task. We ran two LMM with the latency to solve the task as a dependent variable and, as a fixed effect: model 1) the type of test (third-to-last, second-to-last or last learning trial, first or second memory test); and model 2) the retention interval for the memory tests (26–311 days). In these models, the breed, age and sex of the goats were included as fixed factors (control) and the individual identity was fitted as a random factor to control for repeated measurements of the same goats across tests. In model 1, the latency to solve the task was log-transformed in order to satisfy the normal distribution and homoscedasticity assumptions of the LMM. After testing for a general effect of the type of test on the latency to solve the task (model 1), we carried out further tests for two-by-two comparisons. No Bonferroni correction was applied for the posthoc comparisons due to the limited sample sizes [79]. The goat tested during the intermediate memory tests (after 168 days and then another 139 days; goat 4 in Table 1) was not included in the tests carried out on the second memory test because her retention interval was different from the other goats.

Statistical analyses were carried out using R v. 2.9.0 [80]. All tests were two-tailed and the significance level was set at α = 0.05. All means are given with standard errors.

## Competing interests

The authors declare that they have no competing interests.

## Authors’ contributions

EFB, SH and LB collected the data. EFB and SH analysed the data. EFB, SH, LB and AGM designed the study and wrote the manuscript. All authors read and approved the final manuscript.

## Supplementary Material

Additional file 1 **One observer goat (female 7 in Additional file **2**) performing the task on her last training session, followed by her first memory test after a retention interval of 28 days and her second memory test after a retention interval of 281 days.** Click here for file

Additional file 2 Shaping procedure used for the learning phase. Click here for file
