# Supplementary material for: Goats excel at learning and remembering a highly novel cognitive task
Source: Front Zool. 2014 Mar 26;11:20. doi: 10.1186/1742-9994-11-20 (PMC3987177; doi:10.1186/1742-9994-11-20)
Supplement: Additional file 2 — Shaping procedure used for the learning phase. [file 1742-9994-11-20-S2.doc]

**Additional file 2.**  **Shaping procedure used for the learning phase.** Description of stages of the learning phase, along with the corresponding trial numbers, purpose of the stage, duration and action of the experimenter during the trials. The learning criterion was reached when the goats succeeded in obtaining the reward three times in a row for three consecutive trials. The duration of the trials 3-22 was set at 10 min, or until the goats succeeded. The goat numbers mentioned for stage 5 refer to Table 1.

| **Stage** | **Trial number** | **Purpose** | **Duration (min)** | **Action of the experimenter** |
| --- | --- | --- | --- | --- |
| **1** | trial 1 | Habituate to the set-up | 20 | Place food in the bowl after 1 min of distraction |
| **2** | trial 2 | Learn location of reward | 15 | Place food in the bowl after 1 min of distraction |
| **3** | From trial 3 to learning criterion (criterion: minimum = trial 5) | Learn lift-lever step | max 10 | Place food in the bowl and under the lever (trial > 4) after 1min of distraction; refill dispenser after completion |
| **4** | From minimum trial 6 to learning criterion (criterion: minimum = trial 8) | Learn entire task | max 10 | Place food in the bowl and under the lever after 1 min of distraction; refill dispenser and push lever back in after completion |
| **5** | From trial 15 to learning criterion (criterion: range = trial 17-22) | Extra stage for goats 3 and 9: learn entire task | max 10 | Place piece of food around the rope after 1 min of distraction; refill dispenser and push lever back in after completion |
